# Four-Dimensional Printing and Shape Memory Materials in Bone Tissue Engineering

**DOI:** 10.3390/ijms24010814

**Published:** 2023-01-03

**Authors:** Xinwei Zhang, Yixin Yang, Zhen Yang, Rui Ma, Maierhaba Aimaijiang, Jing Xu, Yidi Zhang, Yanmin Zhou

**Affiliations:** 1 Jilin Provincial Key Laboratory of Tooth Development and Bone Remodeling, Hospital of Stomatology, Jilin University, Changchun 130021, China; 2 School of Stomatology, Jilin University, Changchun 130021, China

**Keywords:** 3D printing, 4D printing, bone regeneration, shape memory materials, tissue engineering

## Abstract

The repair of severe bone defects is still a formidable clinical challenge, requiring the implantation of bone grafts or bone substitute materials. The development of three-dimensional (3D) bioprinting has received considerable attention in bone tissue engineering over the past decade. However, 3D printing has a limitation. It only takes into account the original form of the printed scaffold, which is inanimate and static, and is not suitable for dynamic organisms. With the emergence of stimuli-responsive materials, four-dimensional (4D) printing has become the next-generation solution for biological tissue engineering. It combines the concept of time with three-dimensional printing. Over time, 4D-printed scaffolds change their appearance or function in response to environmental stimuli (physical, chemical, and biological). In conclusion, 4D printing is the change of the fourth dimension (time) in 3D printing, which provides unprecedented potential for bone tissue repair. In this review, we will discuss the latest research on shape memory materials and 4D printing in bone tissue repair.

## 1. Introduction

Bone defects caused by congenital deformities, trauma, disease, and surgical resection have become major problem for clinicians [1]. Despite the remarkable self-healing capacity of bone, large-scale irregular bone defect healing remains challenging, especially in the absence of medical intervention [2]. Clinically, the gold standard treatment to resolve such large-scale bone defects consists of filling the defect with an autologous bone graft or allogeneic bone to restore structure and function. However, donor site morbidity and limited bone supply largely limit the efficacy of this treatment [3,4]. Tissue engineering is a promising alternative to bone-deficient or diseased tissue, allowing restoration of affected bone through engineered materials, cells, and growth factors (GFs) [5]. In the past two decades, 3D bioprinting technology for bone tissue engineering has made significant progress, and a large number of studies have combined biomaterials, cells, and bioactive factors to design bone tissue structures and promote bone regeneration through bionic structures [6,7,8,9]. In previous studies, multifunctional 3D printing (3DP) technologies based on different working principles have been applied [2,10,11,12,13]. However, with the deepening understanding of the dynamic biological environment and the continuous improvement of treatment precision requirements, the limitations have prevented traditional 3D printing from meeting the key requirements [14]. In 3D bioprinting, only the initial state of the printed object is considered and it and assumes it is inanimate and static [15], but natural tissue regeneration involves complex 3D structures, microarchitecture, and extracellular matrix components, as well as the generation of tissues with unique functions through dynamic changes in tissues [16,17]. Most of these dynamic functional conformational changes are caused by built-in mechanisms in response to intrinsic or/and extrinsic stimuli that cannot be mimicked by 3D bioprinting [18].

In recent years, shape memory material (SMM) has been proven to be a smart material and has been applied in artificial skin [19], bionic hands [20], bionic flexible joints [21], muscle tissue [22], and bionic soft tongue [23]. Because materials need to respond to external stimuli, these are also known as stimuli-responsive or “smart” materials. Various studies have used a variety of stimuli, including physical (e.g., water [24], temperature [25], light [26], electric and magnetic fields [27]), chemical (e.g., pH [28], and ion concentration [29]) or biological (e.g., glucose and enzymes [30]) stimulation. They can recover their original shape from the temporary shape when exposed to appropriate stimuli [31,32]. In order to further simulate the dynamic in vivo environment, some scholars have found that 4D printing methods can incorporate “time” into current 3D printing by using smart materials [33]. Professor Skylar Tibbits [34] coined the term 4D printing at an MIT conference. He defines 4D printing as “the use of a 3D printer to create objects that can change their form while simultaneously removing them from the 3D printer.” In other words, 4D printing is nothing new but 3D printing with the help of shape memory materials (SMM). This SMM responds to external stimuli and can change shape over time, with the result that it changes specific material properties, resulting in a fourth dimension, time [35]. Currently, most studies are focused on the shape-transforming capabilities of 4D-printed scaffolds, such as folding, lengthening, twisting, and creasing [36]. Over time, shape and function changes of printed structures have been the two main strategies for 4D bioprinting [37]. A series of progressive 4D strategies have been proposed to address current challenges in bone tissue engineering. For example, various stimuli-responsive shape-recovering polymers have been extensively studied as scaffolds and injectable hydrogels suitable for bone tissue engineering [38,39,40]. Their shape memory behavior allows clinicians to implant the printed bone defect model into the body through minimally invasive surgery, and restore it to the ideal initial shape in the body, to achieve minimally invasive surgery in orthopedic treatment [41,42]. The shape-transforming features of 4D-printed bone structures may also meet the needs of personalized bone regeneration, especially in irregular bone defects [43,44]. Furthermore, the functional maturation of 4D-printed bone structures over time may contribute to establishing a biomimetic microenvironment, thereby affecting the cell behavior in the post-printing stage and enhancing the differentiation of stem cells [45,46,47].

However, the application of 4D bioprinting technology and SMM in tissue engineering is still in its infancy, and its concept and mechanism are not widely understood by researchers [48]. In this review, we introduced a series of stimulus-response materials and the application of 4D printing technology in bone tissue engineering, as well as their shape conversion mechanisms for 4D bioprinting. The classification of 4D printing in this review is based on relevant printing technology methods, which opens up an innovative method to manufacture customized implants and personalized accessories, and discusses the challenges faced by 4D biological printing and possible solutions. It is hoped that this review will deepen our understanding of 4D bioprinting in bone tissue engineering.

## 2. The Mechanism and Design Principle of 4D Printing

The 3D printing process is a 3D computer-aided design (CAD) model created by a professional 3D scanner or computer. The 3D model is generally saved as a standard tessellation language (STL) file, then divided into 2D graphics by specialized software, and finally sent to the 3D printer. The printer will print the physical model layer by layer according to the 2D graphics [49]. The emergence of 3D printing provides a good solution for various types of skeletal diseases, including preoperative model construction and scaffolding. At the same time, 3D printing can be used to manufacture scaffolds to solve complex bone defects, and the materials used in 3D printing include metals, polymers, ceramics, and clays [50,51,52]. The printers used for 4D bioprinting are the same as for 3D bioprinting, namely extrusion-based, inkjet-based, and laser-based technologies. Commonly used 3D printing technologies for bone tissue engineering include 3D drawing/direct ink writing (3DP/DIW), stereolithography (SLA), selective laser sintering/selective laser melting (SLM/SLS), fused deposition modeling (FDM) [53,54], and powder printing [55]. 3DP/DIW is based on extruding a viscous material in a syringe to create a 3D shape. The platform remains stationary while the injection head can move, and the extruded material is joined together layer by layer [56]. 3DP requires an additional curing reaction. The advantage of this technique is the flexibility of the resulting material; solutions, slurries, and hydrogels can be loaded into 3DP printers. However, complex structures can collapse due to the low stiffness of the original viscous material. The working principle of the FDM technology is that the thermoplastic filament is fed into the nozzle and heated and melted until it is extruded in a molten state. After the ejected material is cooled rapidly, it solidifies in the platform and follows the trajectory generated by the CAD file to print out 3D solid objects by melting and printing layer by layer [57]. SLS/SLM uses an infrared laser as the energy source to preheat a powder to a predetermined temperature just below its melting point, and then the powder is flattened by a flattening rod. The laser beam is selectively sintered layer by layer according to the layered section information controlled by a computer. After the product is manufactured, all excess powder is removed to obtain a sintered part [58]. SLA uses a liquid photosensitive resin as a raw material, and an ultraviolet (UV) laser beam is controlled by a computer to photocure the surface of the resin to obtain a pattern. For photo-cross-linkable materials, digital light processing (DLP) printers can achieve higher resolution and complex structure [59] (Table 1).

As an extension of 3D printing, 4D printing enables the printed scaffold to change over time [14,15,60]. Compared with 3D printing, the dynamic bioscaffold created by 4D printing can better match the complex physiological environment inside a bone defect. Their unique responsive functionalization and shape transformation capabilities may help to more accurately mimic the dynamics of native orthopedic tissue [55]. However, the definition of 4D bioprinting is still under debate and no consensus has yet been reached. Farhang [60] and Pedro [61] define 4D bioprinting as the use of 3D bioprinting technology to achieve shape, size, or structure changes through stimuli-responsive materials (for example, initially flat structures can evolve into self-folding, flower-like, cube-shaped, or other structures), and biological constructs can be cell-free or cell-lade. Functional transformation and maturation of printed cell-laden structures over time are also considered 4D bioprinting (biological behaviors such as cell fusion, and cell assembly), which both offer unprecedented potential for bone tissue engineering [59].

In 4D bioprinting, the device may be the same as in 3D bioprinting, with the key difference being the material and external stimuli [62]. Moisture-responsive materials absorb or release moisture when exposed to the surrounding environment, causing swelling, twisting, folding, and other deformations [63]. A temperature-responsive material behaves similar to rubber when heated above its glass transition temperature, and upon application of an external force, the material is cooled to a lower temperature and behaves as a rigid solid. The strip bends due to uneven internal thermal stresses within the material. If heated again, the shape memory material returns to its original flat state as it becomes elastic again [64]. The fundamental mechanism of photo-responsive materials is based on the photothermal effect, where light absorbed by a material causes a temperature change [65]. Magnetic field-responsive materials are usually composites of magnetic nanoparticles [27]. When the pH value changes, the polymer chains stretch from spherical to helical, causing the structure to expand, shrink, or bend [28]. Shape memory scaffolds are applied in animals and can restore their desired complex shape when implanted, and can be used in minimally invasive surgery for the regeneration of large bone defects and have the ability to adapt to complex shaped bone defects [66].

## 3. Research Progress of Bone Histology in Various Categories of 3D Printing

### 3.1. DIW

**Figure 1:**
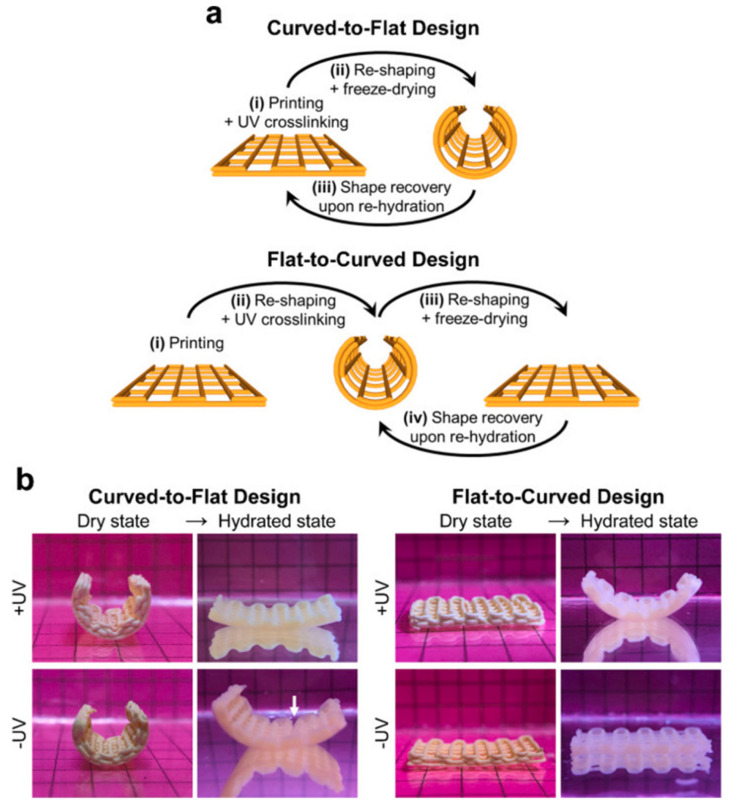
Four-dimensional bioscaffolds with water (humidity) responsiveness by DIW printing technology. Shape memory behavior of printed constructs. (**a**) Schematic illustration of fabrication steps of constructs with shape memory capacity. (**b**) Digital photographs of different construct designs at dry state (temporary geometry) and in the hydrated state showing their shape recovery performance upon immersion in aqueous media. Such deformation characteristics create conditions for minimally invasive implantation of bone grafts. (Reproduced with permission [75]. Copyright 2021, Elsevier).

Direct Ink Writing (DIW) printing technology has relatively mild printing conditions that are conducive to the addition of biomacromolecules and cells. Previously, Luo et al. [67] used 3D-printed alginate/PDA scaffolds to undergo folding shape changes through photothermal effect dehydration under 808 nm laser irradiation. Due to the slow speed of material shape change, the process is easy to control precisely. The speed and degree of shape change can be adjusted by adjusting the laser power and irradiation time. Higher laser powers generate higher temperatures and lead to faster dehydration, resulting in faster shape changes [67]. Crosstalk between bone marrow mesenchymal stem cells (BMSCs) and macrophages plays a crucial role in bone healing. Liu et al. [68] used polyglyceryl sebacate (PGS) and polycaprolactone (PCL) hybrid polymers to print a porous membrane by DIW. Porous membranes coated with polydopamine using GBR technology enabled faster recruitment of M2 macrophages, thereby promoting the recruitment and osteogenic differentiation of BMSCs. At the same time, the 4D memory deformation of the membrane facilitates the repair of alveolar defects around implants [68]. Wang et al. [69] fabricated m-HAp and m-SiO_2_ into scaffolds with multi-scale pores and multifunctional properties using a freeze-drying technique and DIW printing technique. The scaffold not only supported the growth, proliferation, and osteogenic differentiation of mBMSCs, but could also load and slowly release the antibacterial drug enrofloxacin (ENR), which has a long-term antibacterial effect on Escherichia coli and Staphylococcus aureus, which is beneficial to the healing of infected bone tissue [69]. DIW printing technology also has the disadvantage of relatively low manufacturing process precision. Recently, some scholars have developed some new materials for printing complex 3D structures. Zhang et al. [70] designed a novel SMP named poly (glyceryl dodecanoic) acrylate (PGDA) for 4D printing with T_trans_ in the range of room temperature. In addition, the material has excellent rheological properties for the fabrication of various fine 3D structures such as “triangles”, “stars”, “six-petal flowers”, “honeycomb”, and “tubular” shapes. Not only can the scaffold structure have a recovery rate as high as 98% at 37 °C, but more importantly, it has a stable cycle performance of up to 100 cycles and a recovery speed of 0.4 s. Kirillova et al. [71] fabricated hollow self-folding tubes with an average inner diameter as low as 20 µm by DIW. The self-folding tubes consisted of alginate and hyaluronic acid and were double-crosslinked by calcium ions and photocuring. Lai et al. [72] developed deformable hydrogels with swelling anisotropy via alginate (Alg) and methylcellulose (MC), exhibiting excellent rheological properties, extrudability, and shape fidelity of printed structures. The characteristic of this method was that, according to the different expansibility of each part, the printed 2D structure could be transformed into various preset complex 3D forms (for example, coiled tube, helix, double helix, and flower) after being immersed in calcium chloride solution. To improve the mechanical properties of scaffold materials, ceramic-like materials have been selected as reinforcing agents. Wang et al. [44] added a photothermal agent to β-tricalcium phosphate/polylactic acid-trimethylene carbonate (TCP/P(DLLA-TMC)) bioink, printed at low temperature, and also controlled by near-infrared light NIR light deformation. Zhou et al. [73] composed three functional components of fumed silica (FS), liquid metal (LM), and low-boiling point liquid (ethanol) into silicone elastomers, which had good printability and thermal responsiveness. Among them, FS improved the printability of silicone elastomers; LM fillers significantly improved thermal conductivity; and temperature stimulation caused ethanol to evaporate and expand to drive the expansion and deformation of scaffold materials [73]. There are also some studies using DIW technology to promote the shape of cartilage. Ding et al. [74] developed a novel micro flake hydrogel (MFH) with anisotropy for 4D living cell bioprinting ink. MFH’s exhibit desirable shear-thinning, shear-yielding, and fast self-healing properties, and can achieve high resolution and high fidelity without support materials. MFH achieves 4D shape changes under pH-shifting conditions and has been applied to 4D cartilage-like tissue formation (Figure 1).

### 3.2. FDM

PCL and PLA materials are often used as scaffold materials for bone tissue engineering because of their good biocompatibility and degradability. Langford et al. [76] used PLA filaments as raw materials to print bionic bone channels with their herringbone and hydroelastic mosaic origami structures. This design greatly improved the plasticity of the temporary shape and recovered up to 96%. Yue et al. [77] used FDM to print a magnetically responsive Fe_3_O_4_/CNF/PHB/PCL composite scaffold. PLA/CS porous scaffolds printed by Pandey’s method [78] have shown remarkable shape recovery properties and are used as self-healing implants for acute bone defects. Singh et al. [79] used FDM to print temperature-responsive polylactide-graystone. Low-temperature fused filament deposition manufacturing (LFDM) technology does not require high temperatures and can add bioactive macromolecules. Wang et al. [80] added SPIO NPs to PU to improve crystallinity, increase the shape fixation effect, and also to contain polyethylene oxide (PEO) or gelatin to improve printability. The brackets were printed by LFDM and restores their original shape in 50 °C air or 37 °C water. It was observed that the shape memory performance in water was better than in air. The release of SPIO from PU-based scaffolds promoted the secretion and deposition of collagen and calcium from hMSCs in the scaffolds. It is expected to be applied in minimally invasive surgery for bone tissue regeneration [80]. The interaction between neural stem cells and MSCs is beneficial to the functional recovery of bone tissue. For the first time, Miao et al. [81] used complex topographically transformed smart 4D media to enhance the growth and differentiation of neural stem cells in a time-dependent manner. This mimicked the physiological characteristics of NSC neural differentiation and serves as a new culture platform to provide an ideal extracellular microenvironment [81].

### 3.3. SLM

Metals commonly used in bone tissue engineering (e.g., Ni, Ti, AL, Mg.) are difficult to process through conventional 3D printing due to their high melting points. SLM can use high-power lasers to melt metal powders to print metal materials. Among them, bone synthetic materials made of NiTi are often used in minimally invasive surgery of oral and maxillofacial surgery. Moreover, NiTi implants are ideal for advancing minimally invasive and endoscopic procedures. For example, in orbital floor reconstruction, a prefabricated mesh can be rolled or folded for minimally invasive insertion and then self-expand at the target site. Naujokat et al. [82] used the SLM process to print a NiTi scaffold and implanted it in the frontal nasal bone of a pig to verify the biocompatibility of the implant in the bone and subperiosteal. Saedi et al. [83] fabricated a porous NiTi alloy by SLM and achieved an elastic modulus similar to that of cancellous bone by changing different porosity.

### 3.4. DLP

Direct Light Printing (DLP) is a localized photopolymerization technique that is mostly used to print porous or hollow structures. Based on DLP technology, Le Fer et al. [66] used absorbable poly (propylene fumarate) for 3D printing. Cylindrical scaffolds with four different spiral structures with different diameter sizes (140, 200, 240, and 280 μm) and pore sizes (489, 699, 838, and 978 μm) were printed using CAD files. A photocurable silk fibroin (Sil-MA) hydrogel loaded with chondrocytes was printed into an artificial trachea and placed into a rabbit tracheal site with a 210° partial defect [84]. You et al. [85] fabricated a double-layer membrane composed of a hydrogel layer and an SMP layer by DLP printing technology. Due to its flat geometry, the fabricated bilayer membrane can be delivered in vivo by non-invasive means. Once in place, the hydrogel swells and the membrane deforms over time into a predesigned 3D figure in a CAD file. This design can fully wrap geometrically complex bone defects, and has been applied in mouse bone defect models [85].

### 3.5. Electrospinning

Electrospinning has been recognized to produce microfibers/nanofibers with high porosity and specificity and a suitable surface area for various biomedical applications such as tissue engineering, controlled drug delivery, sensing, separation, filtration, and catalysis. Manufactured by double electrospinning, PCL and Pellethane 5863-80A were spun separately and simultaneously to form hybrid fibers. A roller was used for collection, and mouse fibroblasts were used to verify their cytocompatibility [30]. Wang et al. [86] added (3-hydroxybutyrate-co-3-hydroxy valerate) (PHBV) to poly-l-lactide (PLLA) and formed ultrafine composite fibers (PLLA-PHBV) by electrospinning. The composite fibers have >98% shape fixation and >96% shape recovery. In addition, mouse-BMSCs also differentiated toward osteogenesis. Dexamethasone (DEX) is a synthetic glucocorticoid compound and one of the ideal bone formation accelerators with good stability. Lv et al. [87] made microfibers by mixing HA into shape memory polyurethane (SMPU) for electrospinning. Composite fibers can ensure sustained long-term drug release of DEX. The composite fibers were elongated after incubation at 45 °C for 5 min. Samples were then fixed by standing at −20 °C for 30 min. Finally, the temporary shape was placed at 37 °C (close to the physiological temperature of the human body), and the SMPU/3%HA composite fiber could achieve a recovery rate of 93.6% [87].

### 3.6. Others

For the first time, Miao et al. [88] used a novel soybean oil epoxidized acrylate as a material to construct a tissue engineering scaffold using SLA, which is temperature-responsive. The adhesion ability of hMSC on the novel soybean oil epoxidized acrylate sample was significantly higher than that of PEGDA, and similar to that of PLA and PCL [88]. Constante et al. [89] made a complex 4D printing process. The lower layer was 3D printed by a methacrylated alginate gel (AA-MA) hydrogel layer, and the upper layer was the PCL layer of melt electrowetting (MEW). A change in the concentration of Ca 2+ was used as a stimulus for the shape transition, resulting in the formation of a bilayer self-folding tube structure. The tubular structure can also be restored by adding ethylenediaminetetraacetic acid (EDTA) to competitively bind calcium ions [89].

## 4. Research Progress of Shape Memory Materials in Bone Histology

Shape memory material with shape memory effect is considered a smart material that can automatically change size, shape, physical strength, or structure [90]. Usually, the recovery of SMM from a temporary shape to its original shape under external stimuli is the most commonly used method. Its deformation behavior mainly includes folding, unfolding, bending, twisting, surface curling, linear or nonlinear expansion, and contraction [91]. An ideal programmable SMM should have a fast response, reliable recovery rate, ease of fabrication, and high repeatability. In bone tissue engineering, researchers have put forward higher requirements for biomimetic performance, especially reasonable physical strength, heterogeneous structural distribution, and targeted homeostatic regulation in the bone microenvironment [92,93]. Biodegradable SMMs have been widely used in medicine due to their non-toxicity and ability to achieve minimally invasive procedures [94]. Shape memory materials in bone tissue engineering, in addition to enabling minimally invasive surgical implantation, may also offer the possibility of applying in situ mechanical stimulation to enhance the efficacy of bone repair and regeneration. In addition, the application of SMM in tissue engineering has been extended to the induction of related cells, through the regulation of ECM and GFs to promote cell proliferation, directional differentiation and alignment to achieve the goal of promoting tissue repair [95]. Shape memory materials can be classified into shape memory alloys (SMA), shape memory ceramics (SMC), shape memory polymers (SMP), and shape memory hydrogels (SMH) [93] (Table 2). Thus far, shape memory polymers and shape memory hydrogels are the most commonly applied materials for 4D bioprinting because of their good biodegradability and easily tunable mechanical strength [96]. However, in recent years, ceramics and metal compounds have been increasingly used in bone and cartilage tissue engineering because of their inherent advantages in mechanical strength [97]. The performance of a single material is difficult to meet the structural properties of complex bone tissue, and more and more hybrid SMMs with excellent performance have become the first choice [44,98].

### 4.1. Shape Memory Polymer

Shape memory polymers are characterized by their lightweight, reliable shape memory, easy stimulation conditions, biodegradability, low biotoxicity, or even non-toxicity, making them superior to other smart materials [31].

PCL: PCL is an inexpensive FDA-approved polymer and flexible biologic. Despite its biodegradability and biocompatibility, after a large number of long-term experiments researchers found that pure PCL materials degrade slowly and have poor mechanical properties [31]. Erndt-Marino et al. [99] developed a photopolymerizable novel shape memory polymer foam based on poly(ε-caprolactone) diacrylate (PCLDA). These PCLDA foams can be pre-softened with warm saline and implanted in irregular bone defects, and when coated with polydopamine, can induce hydroxyapatite deposition. PD-PCLDA scaffold-loaded human mesenchymal stem cells (h-MSCs) expressed the same levels of Runx2, alkaline phosphatase, and osteopontin when cultured without osteogenic medium supplements in the presence of similar, and inhibited hMSCs to adipose tissue differentiation [99]. R. Pfau et al. [100] used heat-responsive poly (ε-caprolactone)—diacrylate for the treatment of craniomaxillofacial bone defects. Shelby L. Buffington et al. [30] designed a new type of SMP, which had a shape-fixed component PCL that was susceptible to lipase degradation and a shape memory component, Pellethane 5863-80A, that was not susceptible to lipase degradation. Stretching the PCL when the material was heated above the Tm, and then cooling, achieved a temporary shape in which the PCL was in compression and the Pellethane was in tension. Lipase was then added to hydrolyze the ester bond—the PCL was degraded by the enzyme, causing the Pellethane to shrink back to its original shape. One-way and one-shot shape recovery was achieved, leaving the material in its final state [30].

PLA: PLA is a biodegradable polymer composed of lactic acid, made from starch from renewable plants such as sugar cane and corn [114]. Some studies [115] have shown that long-chain branched polylactic acid (LCB-PLA) prepared by solid die drawing technology has multiple memory behaviors and a high recovery rate of 78.8%. However, PLA can only restore a single memory and the recovery rate is lower than 21.5%. After the shape recovery, the mechanical properties of LCB-PLA decreases linearly with the recovery temperature, and as a bone fixation material, it can simultaneously achieve the effect of self-reinforcement and self-fastening [115]. Almost all bones have a section of dense bone, and inside, spongy bones. A tubular structure of PLA was designed, and its surface replaced dense bone with origami herringbone and water bomb mosaic designs. As memory deformation occurred, the cavity in the middle grew, simulating cancellous bone. This design resulted in a large volume difference between the temporary shape and the initial shape, which well mimicked the gap between the trabeculae [76].

PU: Biodegradable shape memory polyurethane has microphase separation and shape memory effect. The resulting ability to promote bone calcification and minimally invasive implantation makes it a promising material for bone repair. Li et al. [116] used PCDL, MDI, and different chain extenders to synthesize biodegradable body temperature-responsive shape memory polyurethane with a self-healing function. By adjusting the chain extender, the transition temperature was close to the body temperature for biomedicine. However, weak mechanical properties hinder its application in bone tissue. To obtain SMPU with higher mechanical properties, Yang et al. [101] first designed and synthesized two extended-chain diisocyanates composed of hexamethylene diisocyanate and isosorbide (ISO), and then used them as coupling. A new type of linear SMPU (ISO-PUs) was synthesized using poly DL-lactic acid (PDLLA)-based macrodiol as the soft segment and ISO as the chain extender, thereby greatly improving the mechanical properties of SMPU and effectively promoting the proliferation of osteoblasts.

PGDA: Poly (glyceryl dodecanoate) acrylate (PGDA) is a new type of SMP that synthesizes PGD from glycerol and dodecanedioic acid and introduces acrylate groups to modify PGD into photocurable PGD, named PGDA. In a mouse aortic graft model, PDGA was successfully used as a material to fabricate vascular grafts to achieve blood circulation, which is expected to be used to improve vascularization in bone tissue [70].

PBF: Guo et al. [102] designed a humidity-responsive SMP poly(butylene fumarate) (PBF) that supports osteoblast attachment, proliferation, and enhanced alkaline phosphatase activity. In addition, PBF can be easily functionalized and loaded with BMP2 via pendant hydroxyl groups for sustained release.

PPF: Poly (propylene fumarate) material synthesized by stepwise polycondensation of diethyl fumarate and propylene glycol exhibits thermal stimulus responsiveness [66].

There are three main fatty acid residues in the novel soybean oil epoxidized acrylates: stearic acid, oleic acid, and linoleic acid, which have pendant alkane groups. At −18 °C, these groups are frozen, which facilitates shape fixation; at 37 °C, the oscillation of these groups facilitates shape recovery and 4D functionality [89].

### 4.2. Shape Memory Hydrogel

Shape memory hydrogels, a class of naturally biodegradable polymers with high biocompatibility, have become common active materials in 4D printing because of their ability to change their volume dramatically in response to stimuli. The disadvantage is that they are weak in mechanical strength, which some studies have improved by material modification or changing the conditions of scaffold synthesis [117]. 

GelMA: Yuan et al. [104] fabricated porous shape memory cryogel microspheres (CMS) from methacrylated gelatin (GelMA) by combining the emulsion technique with gradient cooling cryogels. The pore size of the CMS was adjusted by a gradient cooling procedure, and an optimal pore size (15.5 ± 6.0 µm) was obtained at 30 min gradient cooling (CMS-30). Loading hBMSCs and HUVECs in CMS-30 at a ratio of 1:1 and then subcutaneously injecting them into nude mice successfully promoted the regeneration of vascularized bone tissue.

Gelatin: The nanofibrous gelatin scaffold prepared by Ying et al. [103] through heat-induced phase separation and porogen leaching technology. The scaffold is chemically modified from gelatin with heparin. Heparin can specifically interact with bone morphogenetic protein-2 and make Its stable release. To form injectable, shape memory, highly porous scaffolds, gelatin scaffolds can be injected for bone regeneration of sinus enlargement in rabbits using an external maxillary sinus lift [103]. The gelatin designed by Diba [75] uses UV and humidity to regulate the morphology of 3D-printed scaffolds. 

Collagen: Jiang et al. [105] made Col scaffolds by using type I collagen. Col scaffolds can be fixed into a temporary shape after freeze-drying, followed by shape recovery with moisture. The Col scaffold has the advantage of native collagen, which can enhance chondrocyte adhesion, proliferation, and redifferentiation in the New Zealand white rabbit cartilage defect model.

Alginate: An alginate/polydopamine based bioink was used for cell-loaded printing [67]. In addition, the AA-MA and HA-MA memory deformable hydrogels are cross-linked by calcium ions and alginate, and the microtubules undergo self-folding. When EDTA is added to compete with the calcium ions in the alginate, elongation of the self-folding tubes occurs to restore their original shape, and by loading with mouse bone marrow stromal cells they can differentiate into a variety of cell types, including osteoblasts, chondrocytes, and adipocytes [71]. Hydrogels synthesized from alginate (Alg) and methylcellulose (MC) can also be deformed by calcium ions [72]. 

### 4.3. Shape Memory Alloys

**Figure 2:**
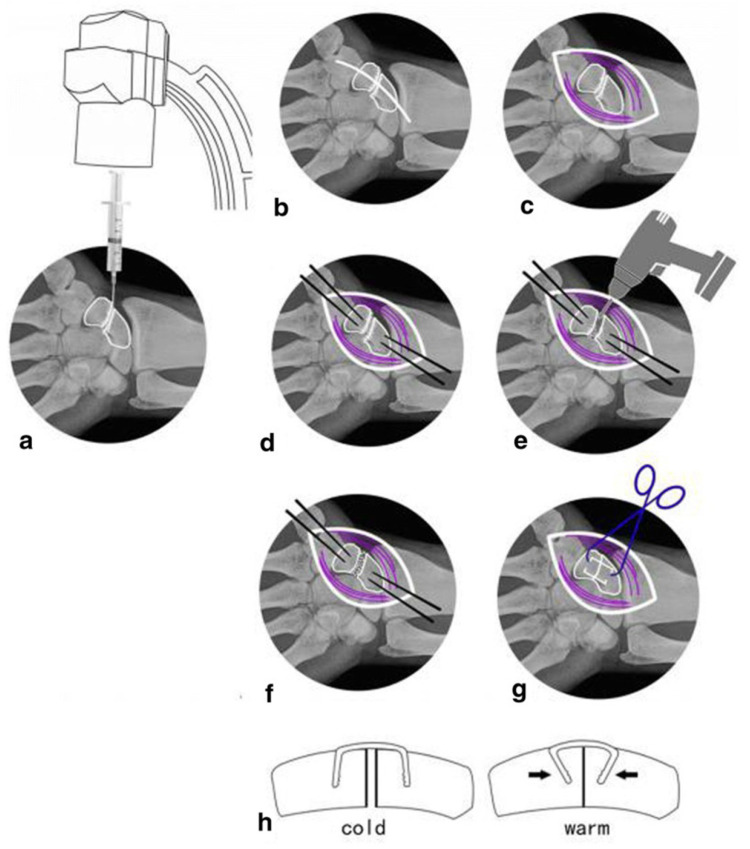
Nitinol dome memory alloy connector used to treat navicular nonunion. (**a**) Puncture the position with a 5ml syringe needle under the fluoroscopy of the c-arm. (**b**,**c**) Cut through skin and muscle tissue (purple) to expose the scaphoid nonunion. (**d**) Determine the appropriate implantation position at the proximal and distal ends of the bone. (**e**) Remove fibrous scar tissue and hardened calluses through electric drilling. (**f**) Repair the cancellous bone graft with attachment cortex. (**g**,**h**) Select a suitable NiTi alloy and insert it, then spray warm (35–40 °C) salt water to gradually tighten the extended arm until it almost reaches its original shape. (Reproduced with permission [118]. Copyright 2019, Springer Nature).

Shape memory alloy (SMA) belongs to the metal alloy system. Due to the high mechanical strength requirements of bone substitutes, scaffolds composed of metal materials are usually used clinically as functional substitutes for natural bone [2]. Cheung et al. [106] conducted the first randomized double-blind clinical trial to investigate the efficacy and safety of a novel superelastic nickel-titanium (NiTi) spinal rod in the treatment of scoliosis in adolescents. Zhou et al. [118] treated scaphoid nonunion with NiTi shape memory alloy connectors combined with autologous iliac bone grafting, providing a new method with less trauma, convenient operation, and satisfactory curative effect for the treatment of scaphoid nonunion (Figure 2). Platelet-rich plasma gel and shape memory nails were used in the treatment of scaphoid nonunion [110]. Roger et al. [107] used NiTi memory alloy combined with autologous bone grafting to treat old nonunion of navicular bone.

However, the mechanical strength mismatch between metal grafts and natural bone can lead to bone resorption and treatment failure, and some scholars have optimized the physical properties of alloys. The Young’s modulus of NiTi alloy can also be controlled by adjusting the porosity size, which can then be matched with bone tissue [83]. Mixed Ti6Al4V and NiTi have been used to make superelastic shape memory alloy bone implants [119]. The Young’s modulus of Ti49.4-Ni50.6 (55.632 GPa) is much lower than that of Ti at 105 GPa and is suitable for orthopedic implants [120]. The β-phase Mg-30 wt%Sc alloy exhibits a shape memory effect and has excellent mechanical properties with an ultimate compressive strength of 603 ± 39 MPa and a compressive strain of 31 ± 3%. Satisfactory osseointegration was observed in animals [113]. New self-expanding shape memory screws were implanted in vertebrae to improve the stability of conventional screws [108]. Hou et al. [111] heat-treated shape memory alloy NiTi rods by an alternating magnetic field to achieve a transformation temperature between 34 and 47 °C and a C-shaped austenite phase. Magnetic induction was used to treat scoliosis in rabbits.

Some memory alloys with special shapes have been designed for use in different parts of the body or for detection. Akbarinia et al. [112] has produced bone-shaped shape memory implants with root fixation capabilities. At −30 °C, the implants are flexible closed-leg cylinders that fit into bone sockets. Once the implant is in contact with the bone cavity, it warms to body temperature and returns to its original bifurcated shape with sufficient stress–strain immobilized to prevent rotational movement. The good primary stability allows shortening of the traditional unloaded bone healing time. Zakaria et al. [109] is implanted under the periosteum of the skull through a device composed of a silicone sheet and a shape memory NiTi alloy strip. Using the principle of periosteal distraction osteogenesis (PDO), a bone is created by lifting the periosteum and soft tissue covered by the shape memory NiTi alloy strip. Growth space, increased vertical bone height, and the invasion of surrounding soft tissue was prevented by the guided bone regeneration (GBR) technique. Srivastava et al. [121] applied shape memory NiTi alloy to fabricate a non-bonding piezoelectric sensor (NBPS) as a good substitute for direct bonded piezoelectric sensor DBPS, and successfully monitored bone injury and healing by using SMA to achieve autonomous operation of the clamping mechanism.

### 4.4. Composite Shape Memory Material

#### 4.4.1. Polymer Plus Polymer

PCL-PLA: PCL and PLA belong to the two most commonly used materials in synthetic polymers, and the viscoelastic transition of PLA/PCL blends occurs at a temperature of about 46 °C, which is nearly 10 °C lower than that of pure PLA. Studies of their biocompatibility make them candidates for use in implantology. By lowering the activation temperature of these PLAs, it is safest to use the material as a precursor for implants. PLA composites containing 10% poly(ε-caprolactone) (PCL) have been used to reduce the activation onset temperature and SME activation energy [122].

PCL-PLLA: Arabiyat et al. [123] designed poly(ε-caprolactone)-diacrylate (PCL-DA) and poly-L-lactic acid (PLLA) hybrid materials. In the absence of osteogenic inducers, the scaffold material can make the expression of osteogenic markers osterix, bone morphogenetic protein-4 (BMP-4); and collagen 1α1 (COL1A1) was increased in h-MSCs.

PU-AT: Self-healing conductive polyurethane scaffolds (PU-AT scaffolds) based on aniline trimer (AT) exhibit excellent shape fixation and shape recovery rates of >98% and >97%, respectively. Notably, the PU-AT scaffold was reported to exhibit excellent self-healing (nearly 95%) at temperatures close to body temperature (40 °C). Furthermore, in terms of cellular behavior for bone tissue engineering, AT-based scaffolds exhibited excellent hASC cell adhesion, proliferation, differentiation, and bone mineralization. At the same time, the expression levels of genes such as RUNX2, COL1, OCN, and ALP were significantly enhanced, consistent with increased extracellular matrix maturation and osteocalcin deposition [124].

PGS-PPS: Xuan et al. [125] designed a temperature-responsive polymer material mixed with poly (glyceryl sebacate) (PGS) and poly (1,3-propylene sebacic acid) (PPS), where the covalent network of PGS is determined in its permanent state, PPS can provide a temporary state with a shape memory function that promotes chondrogenic differentiation while inhibiting osteogenic differentiation of BMSCs in mice.

PGS-PCL: Liu et al. [68] designed a hybrid polymer of PGS and PCL with temperature responsiveness.

#### 4.4.2. Polymer Plus Hydrogel

PLA-Chitosan: Chitosan was added to PLA scaffolds to reduce acidic byproducts, and the porous PLA-TMC/chitosan microspheres prepared by Hu et al. [126] surface and high specific surface area. The addition of chitosan significantly reduced the degradation rate of the microspheres, improved the adhesion and proliferation of MC3T3-E1 cells, and could also enhance ALP activity.

Poly(ε-caprolactone)-diacrylate (PCLDA) and hydrogel bilayer films were used for osteogenesis studies. The MSCs cultured on the film were significantly transferred from the cytoplasm to the nucleus due to the change of mechanical signals, and the nuclear sensitive protein (Lamin A/C) became highly expressed at the nuclear deformation site. All of these enhanced the expression of osteogenic genes in MSCs and then differentiated towards osteogenesis. A higher proportion and more uniform distribution of CD34, RUNX2, and OSX-positive cells were observed in vivo, indicating active angiogenesis and bone healing [85].

PU-gelatin: Superparamagnetic iron oxide nanoparticles (SPIO NPs) have been reported to promote osteogenic induction in h-MSCs. Wang et al. [80] added SPIO to PU/gelatin and PU/PEO scaffolds and seeded hMSCs to evaluate bone regeneration. The SPIO sustained-release effect of the gelatin-polymer hybrid stent was significantly better than that of the polymer-polymer hybrid stent. RUNX2, OCN, and COL I gene markers in hMSCs were also significantly upregulated after 7 and 14 days of induction.

Poly (ethylene glycol) (PEG), alginate, and gelatin derivatives: Hydrogels made with eight-armed PEG-acrylate (PEGA8), and oxidized-and-methacrylated alginate (OMA) by Ding et al. [127] form a double-layer scaffold with GelMA, and the manufacturing parameters such as polymer concentration, UV absorber concentration, UV irradiation time, and hydrogel thickness can be adjusted by adding a photoinitiator to achieve hydraulic coagulation and controllability of glue shape. Simultaneously, multiple cell types (i.e., fibroblasts, stem cells, and cancer cells) wereable to be encapsulated into the bilayer hydrogel scaffolds.

Chitosan biguanide/PANI: The chitosan biguanide/PANI self-healing scaffold material synthesized by Alireza et al. [128] can up-regulate hADSC differentiation, ECM mineralization and maturation, and increase the expression of osteogenic genes RUNX2, COL1 OCN, and ALP.

PU/Gelatin/GelMA: Wu et al. [129] employed PU dispersion, gelatin, and GelMA to fabricate self-healing PUGG hydrogels. The self-healing properties of hydrogels come from the formation of reversible ionic bonds between COO- groups and NH^3+^. Neural stem cells and MSCs have demonstrated cellular crosstalk in DIW-printed PUGG hydrogels.

HA-MA/PCL: Constante et al. [90] fabricated hydrogel and PCL into an ion-responsive double-layer self-folding tube using complex 3D printing techniques.

PLA/CS: Pandey et al. [78] studied the effect of weight percentage of chitosan in PLA, packing density, and stimulation temperature on the recovery rate of the material.

#### 4.4.3. Polymer Plus Ceramics

As the main components of the mineral composition in bone, hydroxyapatite (HA) nanocrystals and calcium phosphate have been widely used as bone fillers and implants for many years due to their excellent bioactivity, biocompatibility, and osteoconductivity [130].

PCL/HA: An PCL/HA scaffold implanted in the mandible of rabbits significantly promoted bone formation around the subperiosteal implant and increased the stability of a titanium implant. Compared with other stents, PCL/HA stents are pre-designed and compressed according to the implanted area, and can be implanted by minimally invasive surgery, return to the original shape after exposure to body temperature, spread to a larger implanted area, and finally, improve the base bone-mass scaffolds [131]. Liu et al. [132] mixed PCL and hydroxyapatite and coated a surface with a layer of calcium alginate and BMP-2 as a material to promote bone repair of mandibular defects in rabbits. Wang et al. [133] synthesized a biomimetic composite scaffold with controllable microporous structure based on poly(ε-caprolactone) (PCL), polytetrahydrofuran (PTMG), and hydroxyapatite, and the obtained scaffold had a variety of pore structures, high connectivity, tunable mechanical properties, and excellent shape-memory properties. The micro-morphology and void structure of the composite scaffolds were controlled by adjusting the HA content for the study of minimally invasive bone repair.

**Figure 3:**
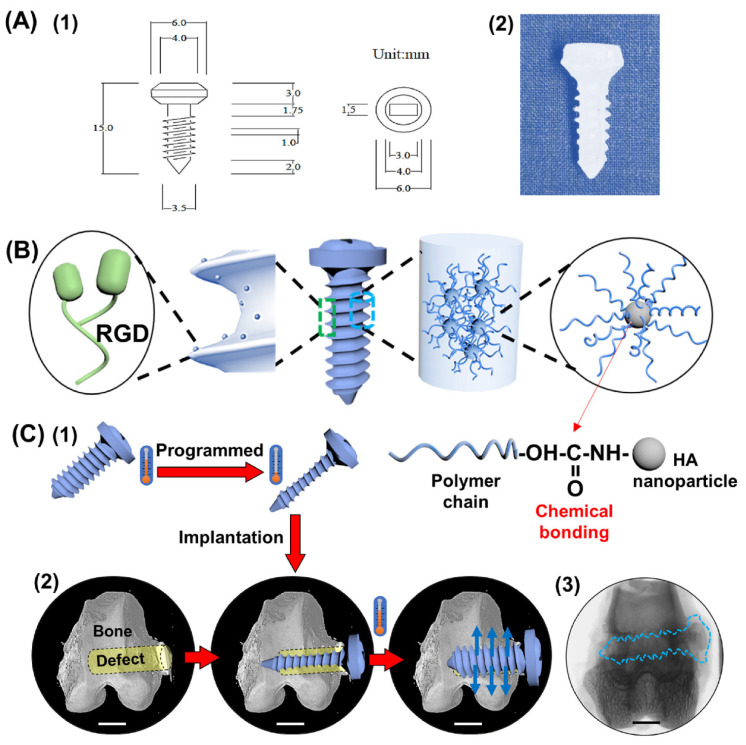
Robust shape memory polymer composite screws made of SMPU/HA/arginylglycylaspartate (RGD) are temperature-responsive to address the stiffness, bioactivity, and internal fixation capabilities of conventional screws limitations. The working schematic diagram of a SMPC screw: (**A**) size (1) and prototype (2) of the bone screw; (**B**) components in the SMPC screw; (**C**) working principle of a memory screw: (1) the screw was compressed at high temperature and then fixed at low temperature; (2) the programmed screw was implanted into the bone defect site by minimally invasive surgery; due to SME, the screw recovered to its original state and offered a supporting effect to the surrounding tissues; blue arrow: recovery force. The scale bars are 5 mm. (3) Representation of a single-slice micro–computed tomography (micro-CT) image after 12 weeks of implantation; blue imaginary line area indicates the SMPC screw. (Reproduced with permission [137]. Copyright 2020, Elsevier).

SMPU/HA: Xie et al. [134] studied the preparation of polyurethane/hydroxyapatite sponge (foam) using a gas foaming method for the treatment of load-bearing bone defects. A rabbit femoral defect model proved that SMP foam expands and deforms through thermal stimulation after implantation to fill bone defects and promote bone growth and neovascularization. Yu et al. [135] used polyurethane/nano-hydroxyapatite (SMPU/nHAP) composite scaffolds for minimally invasive surgery and bone repair. This porous composite scaffold can significantly shorten operation time and promote bone cell growth. Modified SMPU with arginyl-glycyl-aspartate (RGD peptide) and rGO was used to form a cell-adhesive SMPU/HA/rGO/RGD nanocomposite for minimally invasive bone repair by Zhang et al. [136]. The modified nanocomposite showed an approximately 200% increase in Young’s modulus and an over 300% increase in tensile strength over SMPU. The adhesion of rabbit MSCs on composite scaffolds was significantly enhanced. The research group also proposed a robust shape-memory polymer screw made of SMPU/HA/(RGD), HA is osteoinductive and osteoconductive. It has efficient healing and bracing capabilities and is used to address the limitations of conventional screws in terms of stiffness, bioactivity, and internal fixation capabilities [137] (Figure 3). Poly(ε-caprolactone) (PCL)-based polyurethane (PU) microfibers containing hydroxyapatite (HA) exhibit excellent mechanical properties in terms of tensile and modulus values. The test of shape-memory performance found that SMPU and HA composite microfibers exhibited different shape memory transition temperature (T_trans_) values with the addition of HA. After the addition of 3 wt% HA, the fibrous scaffold exhibited excellent shape recovery (>97%) and extremely short recovery time (about 6 s) [88].

PET/HA: Imoto et al. [138] used a polyethylene terephthalate (PET) film as a material to promote bone regeneration and implanted it between the periosteum and bone of the rabbit skull, and the inner side of the film was coated with hydroxyapatite.

TCP/P(DLLA-TMC): Wang et al. [44] added a photothermal agent (BP nanosheets) and an osteoinductive agent (osteogenic peptide) to β-tricalcium phosphate/polylactic acid-trimethylene carbonate) (TCP /P(DLLA-TMC)) bioink for use with low temperature printing by DIW. Through the illumination of near-infrared light (NIR), a composite scaffold with a heat-responsive shape can form shape memory, and can be easily implanted into irregular defects through minimally invasive surgery. Moreover, the release of osteogenic peptide can be controlled by the photothermal effect of NIR to promote the formation of new bone.

Fe_3_O_4_/CNF/PHB/PCL: Yue et al. [77] added Fe_3_O_4_ and cellulose nanofibers (CNF) into polyhydroxybutyrate/poly(ε-caprolactone) (PHB/PCL) mixtures as functional particles. The addition of iron ions endowed the polymer with magnetically responsive shape memory characteristics (Table 3).

## 5. Discussion and Outlook

Four-dimensional bioprinting, which regards “time” as the fourth dimension in 3D bioprinting, is expected to create complex structures with dynamically controllable shape and function, and is considered to be a next-generation tissue engineering technology. In the human body, bone tissue has strong plasticity and has the unique function of adapting to the dynamic changes of the outside world. Traditional 3D-printed scaffolds may have a certain shape or structure but are static throughout. The ultimate goal of 4D bioprinting is to mimic biological functions in vivo as much as possible, and the stimulus conditions that cause changes should be safe and easy to control in vivo. The shape and function transformation features of 4D bioprinting can be used to design and control printed structures with special shape, size, function, or location to meet the requirements of bone tissue engineering and clinical applications. These 4D bioprinting technologies can offer great potential for personalized therapy and minimally invasive medicine.

However, 4D bioprinting is still in the proof-of-concept research stage, and there is still a long way to go to realize the clinical application of bone defects. To date, reliable computational models capable of accurately predicting the deformation of 4D-bioprinted structures are still lacking. Therefore, structural design mainly relies on experimental works and empirical data. It would be more efficient to calculate the dynamic rotation based on the initial structure and external stimuli. According to the mechanical properties, the materials used in 4D bioprinting can be divided into soft materials and hard materials. For soft materials such as hydrogels, biocompatibility is good but mechanical properties are poor. For hard materials, the mechanical properties are good, but the biocompatibility is not satisfactory. Ideal materials for 4D bioprinting should have appropriate stiffness and good biocompatibility. Another requirement for shape memory materials is to respond to complex and diverse stimuli in vivo. However, so far most materials respond to only one stimulus, and most stimulus conditions are not suitable for use in living organisms. Second, existing 4D-printed structures can only undergo simple deformations, such as folding or assembly, which cannot meet the complex needs of clinical bone tissue applications. More efforts should be made in the future to increase the complexity of shape transformation and the precise control of resolution. Repeated folding/unfolding has resulted in significant degradation of the mechanical properties of printed structures. Furthermore, the mechanical strength of printed structures is often not sufficient to withstand high pressures. Therefore, the development of deformable materials that can be repeated multiple times is also a challenge for the future. Issues specific to bone tissue to be considered include optimal internal pore geometry and structure for osteoinductive 4D-printed implants, optimal degradation rate for bone graft substitutes, whether mechanical properties of bone graft substitutes need to mimic native tissue, and how to achieve the vascularization of bone tissue. However, it is believed that with the advancement of material science, printing technology, software, and numerical modeling, 4D bioprinting will take a big step forward in its practical application.

## Figures and Tables

**Table 1:** Multiple classifications for 4D printing.

Printing techniques	SLS, SLM, SLA, DLP, DIW, FDM, LOM, EBM
Smart behaviors	Shape memory, Self-assembly, Self-sensing, Self-healing
Stimulus	Physical (temperature, electrical, magnetic, light)
	Chemical (redox, pH, ion, humidity)
	Biological (glucose, enzyme)
Smart materials/bioinks	Naturally derived polymers (collagen, alginate, gelation)
	Synthetic polymers (PLGA, PLA, PEG, PEO, PVA)

**Table 2:** Different classification of shape memory materials in bone tissue engineering.

Classification	Material	Load	Stimulate	Biological Evaluation	Reference
SMP	PCLDA	PD	Temperature	In vitro: hMSCs	
				In vivo: rabbit femoral defect	[99]
				In vivo: rabbit skull defect	[100]
	PU		Temperature	In vitro: osteoblasts	[101]
	PGDA		Temperature	In vivo: mouse aorta	[70]
	PBF	BMP-2	Water	In vitro: osteoblasts	[102]
	Pellethane		Lipase	In vitro: fibroblasts	[30]
SMH	Gel	BMP-2	Water	In vitro: rabbit BMSCs	
				In vivo:rabbit maxillary sinus	[103]
	GelMA		Temperature	In vitro: hMSCs	
				In vivo:subcutaneous injection	[104]
	Col		Water	In vitro: rabbit chondrocytes	
				In vivo: rabbit knee joint	[105]
	Alginate	PD	Ca^2+^	In vitro: mouse BMSCs	[71]
SMA	Ni-Ti		Temperature	In vivo: adolescent scoliosis	[106]
				In vivo: scaphoid nonunion	[107]
				In vivo: vertebral model	[108]
				In vivo: subperiosteum	[109]
	Ni-Ti	PRP	Temperature	In vivo: scaphoid nonunion	[110]
	Ni-Ti		Magnetic	In vivo: rabbit scoliosis	[111]
	Ni-TiH_2_	Urea	Temperature	In vivo: palatine bone model	[112]
	Mg-Sc		Temperature	In vitro: MC3T3-E1	
				In vivo: rat femoral defect	[113]

**Table 3:** Different hybrid shape memory materials in bone tissue engineering.

Classification	Material	Load	Stimulate	Biological Evaluation	Reference
SMP + SMP	PCL-PLLA	PD	Temperature	In vitro: hMSC	[123]
	PU-AT		Temperature	In vitro: hASC	
				In vivo: rat skull	[124]
	PGS-PPS		Temperature	In vitro: rat BMSC	
				In vivo: rat knee cartilage	[125]
	PGS-PCL	PD	Temperature	In vitro:BMSC, macrophages	
				In vivo: rat skull,	[68]
SMP + SMH	PLA-CS		Temperature	In vitro: MC3T3-E1	[126]
	PCLDA- Gel		Temperature	In vitro: MSC	
				In vivo: rat femur	[85]
	PU-Gel	SPIO	Temperature	In vitro: hMSCs	[80]
	PANI-CS	CP	Temperature	In vitro: hADSCs	[128]
SMP + SMC	PCL-HA		Temperature	In vitro: rabbit BMSCs	
				In vivo: subperiosteal	[131]
		BMP-2	Temperature	In vitro: rabbit BMSCs	
				In vivo: rabbit mandible	[132]
	PU-HA		Temperature	In vitro: MC3T3-E1	
				In vivo: rabbit femur	[134]
		RGD	Temperature	In vitro: rabbit MSC	[136,137]
	PCL/PTMG-HA		Temperature	In vitro: MSC	
	PET-Gel-HA		Temperature	In vivo: subperiosteal	[138]
	PDLLA -TCP	OP	NIR	In vitro: rat BMSC	
				In vivo: rat skull	[44]

## Data Availability

Not applicable.

## Abbreviations

ASC	adipose-derived mesenchymal stem cell
BMP-2	bone morphogenetic protein-2
BMSC	bone marrow mesenchymal stem cells
CS	Chitosan
DIW	direct ink writing
DLP	Direct Light Printing
FDM	fused deposition modeling
Gel	gelatin
GelMA	gelatin methacrylate
HAp	hydroxyapatite particles
PANI	Polyaniline
PCL/PCLDA	poly(ε-caprolactone)/poly(ε-caprolactone)-diacrylates
PD	polydopamine
PGS	poly (glycerol sebacate)
PLA/PLLA/PDLLA	Poly(lactic acid)/poly-L-lactic acid/poly(DL-lactic acid)
PPS	poly (1,3-propylene sebacate)
PU	polyurethane
RGD	arginyl-glycyl-aspartic acid
SLA	stereolithography
SLM/SLS	selective laser melting/ selective laser sintering
SMA/SMC/SMH/SMM/SMP	shape memory alloy ceramics/hydrogel/material/polymer
SPIO	superparamagnetic iron oxide
T_trans_	shape memory transition temperature
